# Pulmonary Thromboembolism in Hospitalized Patients with Acute Exacerbation of COPD: Frequency, Predictors, and D-Dimer Performance

**DOI:** 10.3390/jcm15166394

**Published:** 2026-08-19

**Authors:** Selma Nur Özkiraz Binici, Onur Binici, Sami Deniz

**Affiliations:** 1 Department of Pulmonology, Dr. Suat Seren Chest Diseases and Surgery Training and Research Hospital, University of Health Sciences, 35110 Izmir, Turkey; onurbinici96@gmail.com (O.B.); sami_deniz@yahoo.com (S.D.); 2 The Department of Chest Diseases, Izmir Faculty of Medicine, University of Health Sciences Turkey, 35100 Izmir, Turkey

**Keywords:** acute exacerbation, chronic obstructive pulmonary disease, diagnostic threshold, D-dimer, emergency department, pulmonary thromboembolism

## Abstract

**Background:** Pulmonary thromboembolism (PTE) may mimic or aggravate acute exacerbations of chronic obstructive pulmonary disease (AECOPD). We evaluated the frequency of diagnosed PTE, associated factors, and D-dimer performance in hospitalized patients with AECOPD. **Methods:** In this single-center retrospective study, 429 consecutive patients hospitalized after presenting at the emergency department with AECOPD between 1 June 2022 and 1 June 2023 were screened; 330 met the eligibility criteria. D-dimer testing was performed at the physician’s discretion in 181 patients, who constituted the primary analysis population, and 87 underwent confirmatory imaging. PTE was confirmed by computed tomography pulmonary angiography or ventilation/perfusion scintigraphy. **Results:** PTE was diagnosed in 38 patients, corresponding to 21.0% of the D-dimer-tested population and an observed frequency of 11.5% in the overall eligible cohort. In the primary analysis population, D-dimer showed good discrimination for diagnosed PTE (AUC, 0.826; 95% CI, 0.754–0.898; *p* < 0.001). The exploratory 1053 ng/mL FEU threshold yielded 84.2% sensitivity and 73.4% specificity. In the imaging-confirmed subgroup, the AUC was 0.733 (95% CI, 0.627–0.838), and specificity at this threshold was 55.1%. D-dimer > 1053 ng/mL FEU (adjusted OR, 20.32; 95% CI, 6.80–60.66), female sex (adjusted OR, 4.40; 95% CI, 1.55–12.51), and lower C-reactive protein levels (adjusted OR per 10 mg/L increase, 0.90; 95% CI, 0.83–0.98) were independently associated with diagnosed PTE. **Conclusions:** Diagnosed PTE was frequent among D-dimer-tested hospitalized patients with AECOPD. Because testing and imaging were physician-directed rather than protocolized, the true prevalence in the overall cohort could not be determined. The 1053 ng/mL FEU threshold should be considered exploratory and requires external validation.

## 1. Introduction

Acute exacerbation of chronic obstructive pulmonary disease (AECOPD) constitutes a major cause of emergency department (ED) admissions, leading to substantial healthcare utilization, morbidity, and mortality [1,2,3]. Although respiratory tract infections remain the primary recognized triggers, pulmonary thromboembolism (PTE) is a critical, life-threatening etiology that is frequently overlooked [4,5]. The clinical manifestations of PTE—such as acute worsening of dyspnea, tachypnea, and tachycardia—substantially overlap with the classic symptoms of AECOPD, creating an important diagnostic challenge in emergency settings [6,7]. The reported prevalence of occult PTE in patients experiencing AECOPD varies considerably in the current literature [8,9]. Recent meta-analyses indicate that PTE prevalence can reach 11% to 17% during severe exacerbations [10]. Conversely, large-scale prospective cohort studies utilizing systematic diagnostic algorithms report a lower overall prevalence of 5.9%, which drops to 3.2% even when PTE is not clinically suspected [11]. Despite this variability in prevalence, mortality has been reported to reach 27% when AECOPD is complicated by PTE, compared with 5% in isolated exacerbations. This difference highlights the potential consequences of missed diagnosis and the limitations of relying solely on clinical assessment to safely rule out thromboembolic events [12]. While D-dimer is a high-sensitivity rule-out test when combined with an appropriate assessment of pretest probability, its diagnostic specificity is reduced in the AECOPD cohort. Chronic hypoxemia, underlying systemic inflammation, and the hypercoagulable state associated with chronic obstructive pulmonary disease (COPD) may increase baseline D-dimer concentrations [13,14]. Consequently, applying the conventional rule-out threshold of 500 ng/mL FEU in this specific patient group may yield a high false-positive rate [15]. This diagnostic inefficiency may lead to the overuse of computed tomography pulmonary angiography (CTPA), which burdens the healthcare system and exposes patients to unnecessary contrast-associated kidney injury and radiation risks. Therefore, further evaluation of population-specific D-dimer thresholds may help optimize clinical decision-making [16]. This study aimed to evaluate the frequency of diagnosed PTE among hospitalized patients with AECOPD who underwent physician-directed D-dimer testing, identify clinical and laboratory factors associated with diagnosed PTE, and explore the discriminatory performance of D-dimer and a data-derived threshold in this population.

## 2. Materials and Methods

### 2.1. Study Design and Setting

This retrospective, single-center observational study was conducted at the Dr. Suat Seren Chest Diseases and Surgery Training and Research Hospital, a major tertiary referral center for respiratory diseases in Turkey. The study protocol was formally approved by the local Clinical Research Ethics Committee (Approval Date: 16 August 2023, Decision No: 2023/48-47). The research was conducted in strict adherence to the principles of the Declaration of Helsinki. Due to the retrospective design of the study and the use of anonymized clinical data, the requirement for written informed consent was waived by the institutional ethics committee.

### 2.2. Study Population

We retrospectively screened the electronic medical records of consecutive patients who were hospitalized after presenting to the emergency department with a primary diagnosis of AECOPD between 1 June 2022 and 1 June 2023. AECOPD was defined as an acute worsening of respiratory symptoms in accordance with internationally recognized criteria [3].

Patients were eligible for the overall hospitalized cohort if they had an established diagnosis of COPD, presented with an acute exacerbation requiring hospitalization, and had sufficient clinical data available for assessment. To minimize the influence of conditions that could independently elevate D-dimer levels or strongly predispose to thromboembolism, patients meeting any of the following criteria were excluded: hospitalization within the preceding three months because of lower-extremity fracture or congestive heart failure; atrial fibrillation or flutter; recent major orthopedic surgery; major trauma; myocardial infarction within the preceding three months; active malignancy; spinal cord injury; a documented history of venous thromboembolism; or current therapeutic anticoagulant use.

After application of these criteria, 330 patients constituted the eligible hospitalized cohort. Among these patients, D-dimer testing was ordered at the discretion of the treating physician in 181 patients, who constituted the primary analysis population. The remaining 149 patients did not undergo D-dimer testing and were included only to describe the overall patient flow and real-world utilization of D-dimer testing; they were not included in analyses evaluating D-dimer performance or factors associated with diagnosed PTE.

### 2.3. Data Collection and Diagnostic Classification

Clinical and demographic data were systematically extracted from the hospital electronic registry. Baseline variables included age, biological sex, smoking exposure in pack-years, comorbidities, and long-term oxygen therapy. Pulmonary function test parameters obtained during a clinically stable period included FEV1% predicted, FVC% predicted, and the FEV1/FVC ratio.

Laboratory parameters measured upon index emergency department presentation included D-dimer, C-reactive protein, white blood cell count, pro-brain natriuretic peptide, troponin T, and routine biochemical variables. Plasma D-dimer concentrations were measured using the VIDAS D-Dimer Exclusion II assay on a VIDAS platform (bioMérieux, Marcy-l’Étoile, France), an automated quantitative enzyme-linked fluorescent assay (ELFA), and were reported in ng/mL fibrinogen-equivalent units (FEU). According to the institutional laboratory reporting system, the reference threshold was <500 ng/mL FEU for patients aged 50 years or younger and <age × 10 ng/mL FEU for patients older than 50 years.

D-dimer testing and subsequent imaging were performed as part of routine clinical care according to the treating physician’s clinical assessment; no predefined study protocol or standardized study-mandated diagnostic algorithm was applied. The diagnosis of PTE was confirmed by computed tomography pulmonary angiography or ventilation/perfusion scintigraphy.

Among patients who underwent D-dimer testing, those with imaging-confirmed PTE were classified as having diagnosed PTE. Patients without a documented PTE diagnosis during the index hospitalization were classified as having no diagnosed PTE in the primary real-world analysis. This latter group included both patients in whom PTE was excluded by imaging and patients who did not undergo confirmatory imaging. Patients without imaging were therefore not considered imaging-confirmed negative cases.

### 2.4. Outcomes

The primary outcome was the frequency of diagnosed PTE among hospitalized patients with AECOPD who underwent D-dimer testing during emergency department evaluation.

Secondary outcomes included the frequency of diagnosed PTE in the overall eligible hospitalized cohort; comparison of clinical and laboratory characteristics between patients with and without diagnosed PTE; assessment of the discriminatory performance of D-dimer; identification of factors independently associated with diagnosed PTE; and evaluation of D-dimer performance in a sensitivity analysis restricted to patients with imaging-confirmed PTE status.

### 2.5. Statistical Analysis

All statistical analyses were performed using IBM SPSS Statistics for Windows, Version 26.0 (IBM Corp., Armonk, NY, USA). Distributional normality was assessed using the Kolmogorov–Smirnov and Shapiro–Wilk tests, as appropriate. Continuous variables were presented as the mean ± standard deviation or median with interquartile range according to their distribution. Categorical variables were expressed as numbers and percentages.

Comparisons between patients with and without diagnosed PTE within the primary analysis population were performed using Student’s *t*-test or the Mann–Whitney U test for continuous variables and the Pearson chi-square test for categorical variables. Comparisons between patients who underwent D-dimer testing and those who did not were performed using the same statistical methods and are presented as a supplementary analysis.

Receiver operating characteristic curve analysis was used to evaluate the discriminatory performance of D-dimer for diagnosed PTE in the primary analysis population. The exploratory optimal threshold was determined using the Youden index. Because confirmatory imaging was not performed in all patients, an additional sensitivity analysis was conducted among patients with imaging-confirmed PTE status. In this subgroup, the sensitivity, specificity, positive predictive value, and negative predictive value of the conventional 500 ng/mL FEU threshold, the age-adjusted institutional threshold, and the exploratory 1053 ng/mL FEU threshold were calculated with 95% confidence intervals. The 95% confidence intervals for sensitivity, specificity, positive predictive value, and negative predictive value were calculated using the Wilson score method.

Variables considered clinically relevant to PTE were initially evaluated using univariable logistic regression. Variables with *p* < 0.05 in univariable logistic regression were considered for inclusion in the multivariable model. Age was also retained because of its potential clinical relevance and confounding effect, irrespective of its univariable statistical significance. Because FEV1% predicted, FVC% predicted, and the FEV1/FVC ratio are closely related spirometric indices, only FEV1% predicted was included in the final model as the principal measure of airflow-limitation severity. D-dimer was entered into the model as a binary variable using the exploratory threshold of 1053 ng/mL FEU. CRP was divided by 10 so that the resulting odds ratio represented the effect associated with each 10 mg/L increase.

The final multivariable model included all 181 patients, with no missing data for the included variables. Multicollinearity was evaluated using tolerance and variance inflation factor values. Linearity in the logit for continuous predictors was assessed using the Box–Tidwell procedure. Because the Box–Tidwell procedure indicated nonlinearity in the logit for smoking exposure, its linear univariable odds ratio was not reported, and it was not included in the multivariable model. Model calibration was evaluated using the Hosmer–Lemeshow goodness-of-fit test. Results were reported as odds ratios with 95% confidence intervals. A two-sided *p* value < 0.05 was considered statistically significant.

## 3. Results

### 3.1. Study Population, Baseline Characteristics, and Frequency of Diagnosed PTE

**Figure 1:**
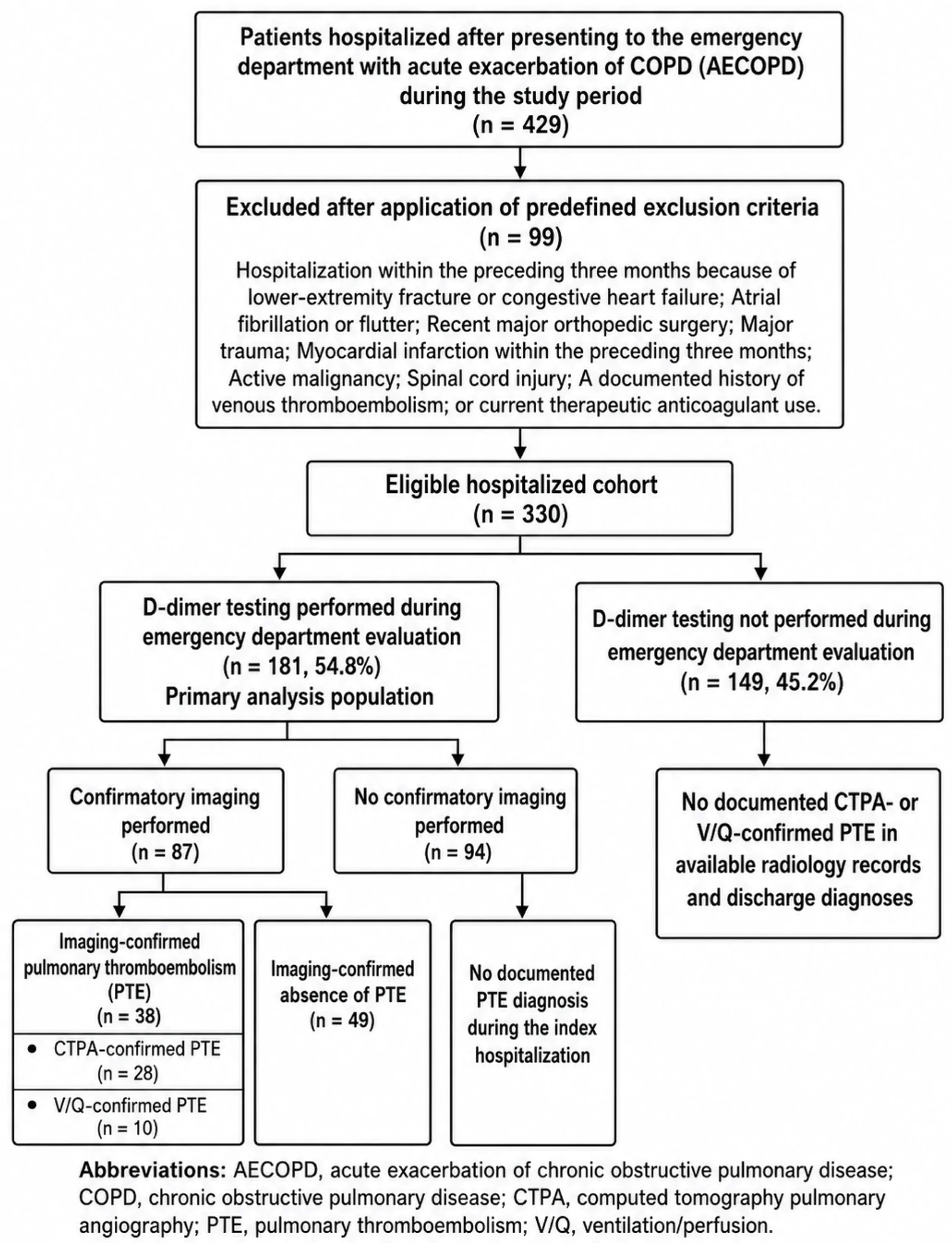
Flow chart of patient selection.

During the study period, 429 consecutive patients hospitalized after presenting to the emergency department with AECOPD were screened. After application of the predefined exclusion criteria, 330 patients constituted the eligible hospitalized cohort (Figure 1).

D-dimer testing was performed in 181 patients (54.8%), who formed the primary analysis population, whereas 149 patients (45.2%) did not undergo D-dimer testing during the emergency department evaluation.

Available stable-period spirometric parameters were compared exploratorily according to D-dimer testing status. Patients who underwent D-dimer testing had significantly higher FEV1% predicted, FVC% predicted, and FEV1/FVC values than those who did not undergo testing (Supplementary Table S1).

In the primary analysis population, the mean age was 69 ± 8 years, and 50 patients (27.6%) were female. Sixty-five patients (35.9%) were receiving long-term oxygen therapy. The most common comorbidities were hypertension in 71 patients (39.2%), diabetes mellitus in 48 (26.5%), coronary artery disease in 38 (21.0%), and congestive heart failure in 28 (15.5%).

Among the 181 patients who underwent D-dimer testing, 87 underwent confirmatory imaging, including 72 CTPA examinations and 15 V/Q scans. PTE was confirmed in 38 patients, including 28 diagnoses by CTPA and 10 by V/Q scintigraphy, whereas imaging excluded PTE in 49 patients. The remaining 94 patients did not undergo confirmatory imaging and had no documented diagnosis of PTE during the index hospitalization. Accordingly, diagnosed PTE accounted for 21.0% of the primary analysis population and 11.5% of the overall eligible hospitalized cohort.

Among the 149 patients who did not undergo D-dimer testing, review of the available radiology records and discharge diagnoses identified no imaging-confirmed PTE. However, because these patients were not evaluated using a standardized diagnostic protocol, PTE could not be considered definitively excluded in this subgroup.

Baseline clinical and laboratory characteristics were compared between patients with and without diagnosed PTE (Table 1). Patients without diagnosed PTE had significantly higher CRP levels than those with diagnosed PTE (*p* = 0.021). Stable-period pulmonary function was also more impaired among patients without diagnosed PTE, as reflected by lower FEV1% predicted (*p* < 0.001), FVC% predicted (*p* = 0.009), and FEV1/FVC (*p* = 0.002) values.

### 3.2. Laboratory Findings According to Diagnosed Pulmonary Thromboembolism Status

D-dimer (ng/mL FEU) levels were significantly higher in patients with diagnosed PTE than in those without diagnosed PTE [1486 (1179–2590) vs. 696 (456–1123), *p* < 0.001].

Inflammatory markers were lower among patients with diagnosed PTE. Median CRP levels were 24 (8–46) mg/L in patients with diagnosed PTE and 45 (10–125) mg/L in those without diagnosed PTE (*p* = 0.021). White blood cell counts were also lower in the diagnosed PTE group [9 (7–10) × 10^3^/µL vs. 10 (8–13) × 10^3^/µL; *p* = 0.041].

No statistically significant differences were observed between the groups in arterial blood gas parameters, urea, creatinine, platelet count, troponin T, or pro-BNP levels. Pulmonary function and laboratory findings according to diagnosed PTE status are summarized in Table 2.

### 3.3. Diagnostic Performance of D-Dimer

**Figure 2:**
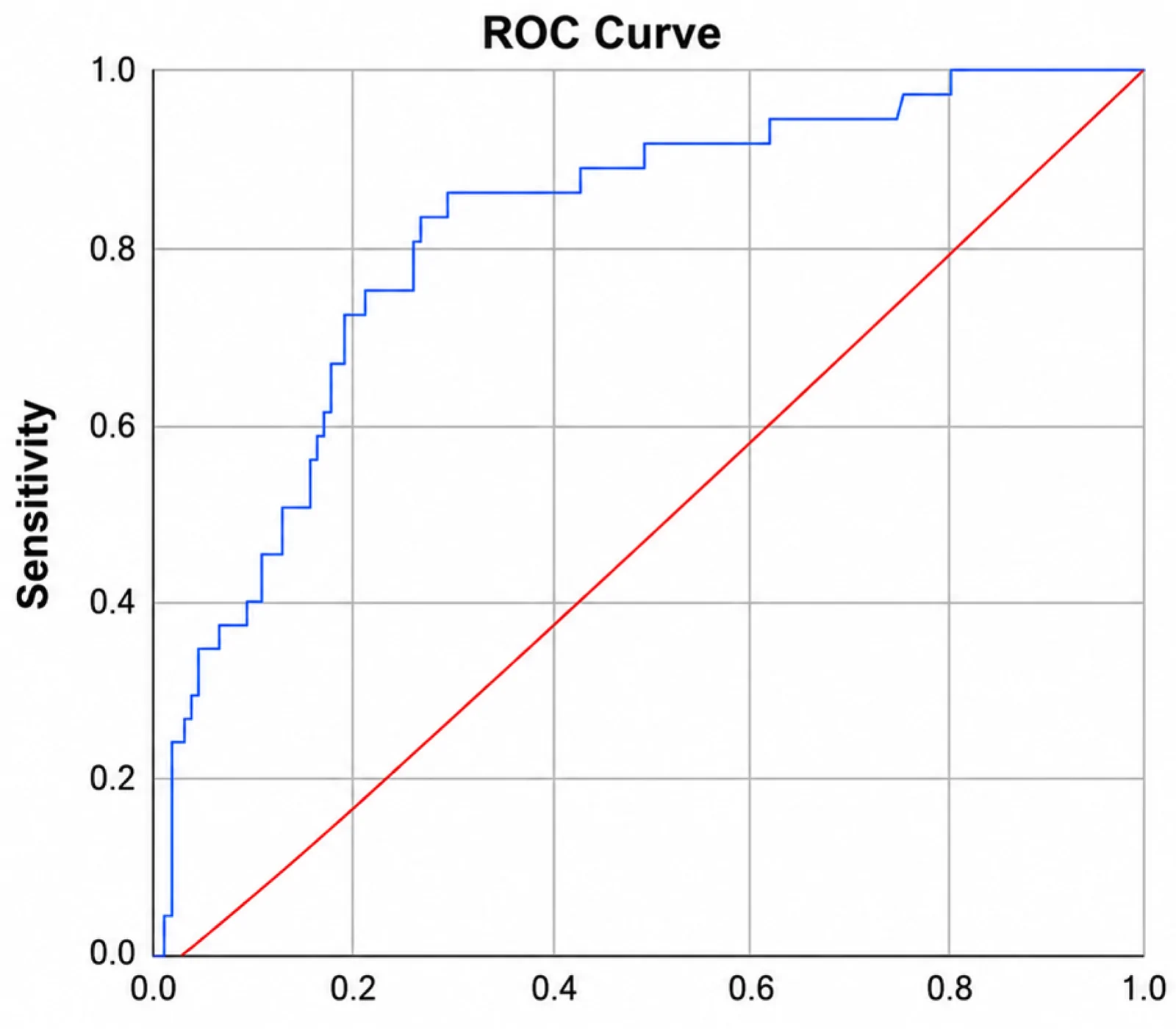
Receiver operating characteristic curve of D-dimer for diagnosed PTE in the primary analysis population (*n* = 181). The area under the curve was 0.826 (95% CI, 0.754–0.898; *p* < 0.001). The exploratory optimal cut-off was 1053 ng/mL FEU, with a sensitivity of 84.2% and a specificity of 73.4%. The blue line represents the ROC curve, and the red diagonal line represents the reference line of no discrimination.

In the primary analysis population (*n* = 181), receiver operating characteristic (ROC) curve analysis showed that D-dimer discriminated patients with diagnosed PTE from those without diagnosed PTE, with an area under the curve (AUC) of 0.826 (95% CI, 0.754–0.898; *p* < 0.001). The optimal D-dimer cut-off determined using the Youden index was 1053 ng/mL FEU, providing a sensitivity of 84.2% and a specificity of 73.4%. The ROC curve for the primary analysis population is shown in Figure 2.

A sensitivity analysis was performed among the 87 patients with imaging-confirmed PTE status. In this subgroup, the AUC for D-dimer was 0.733 (95% CI, 0.627–0.838; *p* < 0.001). At the conventional threshold of 500 ng/mL FEU, sensitivity was 94.7% and specificity was 16.3%. The age-adjusted threshold yielded a sensitivity of 92.1% and a specificity of 36.7%, whereas the exploratory threshold of 1053 ng/mL FEU yielded a sensitivity of 84.2% and a specificity of 55.1%. The diagnostic performance of these thresholds is presented in Table 3.

### 3.4. Factors Independently Associated with Diagnosed Pulmonary Thromboembolism

In univariable logistic regression analyses, D-dimer > 1053 ng/mL FEU, female sex, higher FEV1% predicted, higher FVC% predicted, a higher FEV1/FVC ratio, and lower CRP levels were associated with diagnosed PTE.

The final multivariable logistic regression model included D-dimer > 1053 ng/mL FEU, sex, CRP, age, and FEV1% predicted. D-dimer > 1053 ng/mL FEU was strongly associated with diagnosed PTE (adjusted OR, 20.32; 95% CI, 6.80–60.66; *p* < 0.001). Female sex was also independently associated with diagnosed PTE (adjusted OR, 4.40; 95% CI, 1.55–12.51; *p* = 0.005). Each 10 mg/L increase in CRP was associated with lower odds of diagnosed PTE (adjusted OR, 0.90; 95% CI, 0.83–0.98; *p* = 0.015). Age (adjusted OR, 0.99; 95% CI, 0.94–1.04; *p* = 0.722) and FEV1% predicted (adjusted OR, 1.023; 95% CI, 0.995–1.052; *p* = 0.113) were not independently associated with diagnosed PTE.

The model included all 181 patients, with no missing data for the included variables. No problematic multicollinearity was detected (tolerance range, 0.861–0.947; variance inflation factor range, 1.056–1.161). The Box–Tidwell procedure showed no evidence of nonlinearity in the logit for age, CRP per 10 mg/L increase, or FEV1% predicted (all *p* > 0.05). The overall model was statistically significant (χ^2^ = 69.279, df = 5; *p* < 0.001) and demonstrated adequate calibration according to the Hosmer–Lemeshow goodness-of-fit test (χ^2^ = 2.810, df = 8; *p* = 0.946). Univariable and multivariable logistic regression analyses are presented in Table 4.

## 4. Discussion

In this single-center retrospective study of hospitalized patients presenting with AECOPD, PTE was diagnosed in 21.0% of patients who underwent D-dimer testing during emergency department evaluation and in 11.5% of the overall eligible hospitalized cohort. The main findings were that diagnosed PTE was frequent among D-dimer–evaluated patients, D-dimer demonstrated good discriminatory performance for diagnosed PTE in the primary analysis population, and an exploratory D-dimer threshold >1053 ng/mL FEU, female sex, and lower CRP levels were independently associated with diagnosed PTE.

The frequency of diagnosed PTE in our primary analysis population was higher than the 5.9% prevalence reported in the PEP study [11], in which PTE was systematically evaluated among hospitalized patients with COPD and acutely worsening respiratory symptoms. This difference is likely related to the design of our study, because the primary analysis was restricted to patients who underwent D-dimer testing in routine emergency department practice. Therefore, the 21.0% frequency observed in this subgroup should not be interpreted as the true prevalence of PTE among all hospitalized patients with AECOPD. Instead, it reflects the frequency of diagnosed PTE among patients selected for D-dimer testing in a real-world clinical setting.

When the eligible hospitalized cohort was considered as a whole, the observed diagnosed PTE frequency was 11.5%, which was numerically closer to the pooled estimates reported in previous meta-analyses. Han et al. [17] reported an overall PTE prevalence of approximately 11% among patients with AECOPD, while Fu et al. [10] emphasized that reported prevalence varies substantially according to patient selection, clinical setting, and diagnostic strategy. This heterogeneity is particularly important when interpreting retrospective studies because the observed frequency depends not only on disease burden but also on how actively PTE is investigated. Because D-dimer testing and confirmatory imaging were not performed systematically in the entire eligible cohort, the 11.5% frequency may underestimate the true prevalence of PTE.

This concern is also reflected in the Lancet Commission on COPD, which drew attention to pulmonary embolism as a clinically important condition that may accompany or mimic COPD exacerbations. The Commission noted that, among patients with COPD who present with worsening dyspnea, the prevalence of pulmonary embolism may reach approximately 20% [18,19]. It also emphasized that the absence of a standardized approach to ruling out alternative diagnoses during exacerbation assessment may lead to diagnostic misclassification and inappropriate treatment. In this context, our findings reinforce the importance of considering PTE in hospitalized patients with AECOPD, especially when the severity of dyspnea appears disproportionate to infectious or inflammatory findings [18].

Beyond this diagnostic overlap, several mechanisms may contribute to the development of PTE in patients with COPD, even in the absence of major conventional provoking factors [20]. Acute exacerbations may induce a prothrombotic milieu through systemic inflammation, endothelial dysfunction, platelet activation, and activation of the coagulation cascade. In addition, hypoxemia, reduced mobility during acute illness, and the high burden of cardiovascular comorbidities may further increase thromboembolic risk [21,22]. Collectively, these pathophysiological mechanisms may explain why PTE frequently accompanies or mimics AECOPD and underscore the importance of maintaining a high index of clinical suspicion, even in patients without identifiable secondary provoking factors.

An important aspect of our study is that D-dimer testing was not performed in nearly half of the eligible hospitalized cohort. In routine emergency department practice, D-dimer testing was performed according to the attending physician’s clinical assessment rather than a standardized study protocol. The decision not to perform D-dimer testing likely reflected a lower clinical suspicion for PTE, with alternative causes of exacerbation being considered more prominent during the initial evaluation. Review of available radiology records and discharge diagnoses did not identify any CTPA- or V/Q-confirmed PTE among these patients. Nevertheless, because a standardized diagnostic algorithm was not applied to all patients, the absence of documented PTE in the non-tested subgroup should not be interpreted as definitive exclusion. Moreover, the selective use of confirmatory imaging introduced partial verification bias and potential outcome misclassification, particularly in the primary ROC analysis. In the PEP study, VTE was detected not only in patients with suspected PTE but also in patients in whom PTE was not initially suspected, suggesting that clinical assessment alone may not identify all cases in hospitalized COPD patients [11]. Similarly, a post-hoc analysis of the PEP trial highlighted the ongoing uncertainty regarding optimal PTE diagnostic strategies in AECOPD and the need to balance unnecessary imaging against the risk of missing clinically relevant events [23]. Therefore, our findings should be interpreted as real-world data reflecting physician-directed diagnostic evaluation, while also underscoring the need for prospective studies using standardized algorithms.

The clinical profile of patients with diagnosed PTE in our cohort is noteworthy. Patients with diagnosed PTE had lower CRP and white blood cell counts than those without diagnosed PTE, while stable-period spirometric parameters were relatively better preserved. This pattern may suggest that PTE should be considered particularly in hospitalized AECOPD patients whose clinical deterioration is not strongly supported by infectious or inflammatory markers. In addition, chemoreflex dysfunction in chronic pulmonary disease may alter ventilatory responses, further limiting the specificity of dyspnea severity for distinguishing PTE from AECOPD [24]. Conversely, patients with higher inflammatory markers and more advanced airflow limitation may have exacerbations more readily attributed to infectious or inflammatory decompensation. The better-preserved spirometric findings should not be interpreted as indicating that preserved lung function increases the risk of PTE; rather, they may reflect clinical selection, whereby PTE is more readily investigated when acute deterioration appears disproportionate to the severity of baseline airflow limitation. This interpretation is consistent with recent studies suggesting that exacerbation phenotype, including sputum purulence and inflammatory presentation, may help refine the clinical suspicion of PTE in AECOPD [25]. The inverse association with CRP may reflect a clinical phenotype characterized by less prominent systemic inflammation; however, residual confounding and clinical selection should also be considered when interpreting this finding.

D-dimer was the strongest factor independently associated with diagnosed PTE in our cohort. Receiver operating characteristic analysis identified an optimal cut-off value of 1053 ng/mL FEU, which showed a sensitivity of 84.2% and a specificity of 73.4% in the primary analysis population. In the imaging-confirmed subgroup, this threshold retained a sensitivity of 84.2% but had a specificity of 55.1%, whereas the conventional and age-adjusted thresholds provided higher sensitivity but lower specificity. This finding is clinically relevant because D-dimer interpretation in AECOPD is challenging. A negative D-dimer may help exclude PTE in appropriate clinical probability settings, but positive results are common in acute inflammatory or hospitalized medical conditions and may reduce test specificity [26]. Previous studies have also suggested that D-dimer levels may be elevated during AECOPD even in the absence of PTE, limiting the utility of conventional thresholds in this population [13,15]. Therefore, the 1053 ng/mL FEU threshold identified in our study should be interpreted as an exploratory, population-specific value. Because it was derived and evaluated in the same cohort, its performance may have been overestimated, and it should not be used as a standalone diagnostic threshold before prospective external validation.

Female sex was also independently associated with diagnosed PTE in our multivariable model. Although this finding is clinically interesting, it should be interpreted cautiously. The number of PTE events was limited, and residual confounding cannot be excluded. Previous meta-analytic data have not consistently established sex as a robust predictor of PTE in AECOPD; therefore, this result should be considered hypothesis-generating rather than definitive [17].

Although FEV_1_%, FVC%, and FEV_1_/FVC ratio were significantly lower in patients without diagnosed PTE in univariable comparisons, only FEV_1_% was included in the final multivariable model to avoid simultaneously including closely related spirometric indices. FEV_1_% did not remain independently associated with diagnosed PTE after adjustment. This suggests that better-preserved pulmonary function may help characterize the clinical phenotype of patients with PTE in unadjusted analyses, but it should not be interpreted as an independent predictor in our cohort.

This study has several limitations. First, it was retrospective and single-center, which limits generalizability. Second, the cohort included only hospitalized patients and therefore primarily reflects moderate-to-severe exacerbations requiring inpatient treatment; patients with mild exacerbations discharged from the emergency department were not included. Third, D-dimer testing and subsequent imaging were performed as part of routine clinical care rather than according to a standardized study-mandated diagnostic algorithm. Therefore, our findings reflect physician-directed real-world diagnostic practice rather than systematic screening. Although review of available radiology records and discharge diagnoses did not identify confirmed PTE among patients without D-dimer testing, the true prevalence of PTE in the entire eligible cohort could not be determined. Because confirmatory imaging was performed in 87 of the 181 patients in the primary analysis population, partial verification bias and potential outcome misclassification cannot be excluded. To address this issue, we additionally evaluated D-dimer performance in the subgroup with imaging-confirmed PTE status. The 1053 ng/mL FEU D-dimer threshold was derived and evaluated in the same cohort and should therefore be regarded as exploratory pending external validation. Finally, although the multivariable model was restricted to five clinically relevant variables and demonstrated adequate calibration, the relatively limited number of PTE events warrants cautious interpretation because some degree of model overfitting remains possible.

Despite these limitations, the study provides clinically relevant real-world data. It shows that diagnosed PTE is frequent among D-dimer–evaluated hospitalized patients with AECOPD and suggests that reliance on routine clinical judgment alone may not identify all cases. The association of PTE with higher D-dimer levels and lower inflammatory markers may help identify patients in whom thromboembolic disease should remain in the differential diagnosis. Prospective multicenter studies using standardized diagnostic algorithms are needed to clarify the true burden of PTE in hospitalized AECOPD patients and to validate clinically useful D-dimer thresholds in this population.

## 5. Conclusions

In this single-center retrospective study of hospitalized patients with acute exacerbation of COPD, diagnosed PTE was identified in 21.0% of patients who underwent D-dimer testing and in 11.5% of the eligible hospitalized cohort. D-dimer > 1053 ng/mL FEU, female sex, and lower CRP levels were independently associated with diagnosed PTE.

Because D-dimer testing and subsequent imaging were not performed according to a standardized diagnostic algorithm, and because patients without D-dimer testing were not systematically evaluated for PTE, the true prevalence of PTE in the overall hospitalized cohort could not be determined. Nevertheless, the observed frequency of diagnosed PTE among D-dimer–evaluated patients suggests that thromboembolic disease should remain an important differential diagnosis in hospitalized AECOPD patients, particularly when clinical deterioration is accompanied by elevated D-dimer levels and less prominent systemic inflammatory findings.

The D-dimer threshold identified in this study should be interpreted as exploratory and requires prospective external validation before routine clinical implementation. Future multicenter studies using standardized diagnostic algorithms are needed to clarify the true burden of PTE in hospitalized patients with AECOPD and to determine which patients may benefit most from systematic diagnostic evaluation.

## Figures and Tables

**Table 1:** Baseline clinical characteristics according to diagnosed PTE status in the primary analysis population.

Variables	Category	Diagnosed PTE (*n* = 38)	No Diagnosed PTE (*n* = 143)	*p* Value
Age, years		71 ± 10	68 ± 8	0.105
Sex, *n* (%)	Male	19 (50.0)	112 (78.3)	**0.001**
	Female	19 (50.0)	31 (21.7)	
Smoking status, *n* (%)	Never	7 (18.4)	19 (13.3)	0.490
	Former smoker	16 (42.1)	53 (37.1)	
	Current smoker	15 (39.5)	71 (49.7)	
Smoking exposure, pack-years ^a^		35 (10–55)	50 (30–60)	**0.021**
Long-term oxygen therapy, *n* (%)		10 (26.3)	55 (38.5)	0.165
Diabetes mellitus, *n* (%)		10 (26.3)	38 (26.6)	0.974
Hypertension, *n* (%)		18 (47.4)	53 (37.1)	0.247
Coronary artery disease, *n* (%)		6 (15.8)	32 (22.4)	0.375
Congestive heart failure, *n* (%)		6 (15.8)	22 (15.4)	0.951

Categorical variables are presented as *n* (%). If the numeric variables fitted the normal distribution, they are presented as the mean ± SD; if not, they are presented as the median (IQR 25, 75) in this table. PTE, pulmonary thromboembolism. ^a^ Smoking-exposure data were available for 171 patients: 37 patients with diagnosed PTE and 134 patients without diagnosed PTE. Bold values indicate statistical significance (*p* < 0.05).

**Table 2:** Pulmonary function, arterial blood gas, and laboratory findings according to diagnosed PTE status in the primary analysis population.

Variable	Diagnosed PTE (*n* = 38)	No Diagnosed PTE (*n* = 143)	*p* Value
FEV_1_, L	1.235 (0.94–1.57)	1.05 (0.80–1.38)	0.054
FEV_1_, % predicted	56.26 (38.68–69.14)	40.24 (31.28–49.51)	**<0.001**
FVC, L	2.04 (1.60–2.73)	1.94 (1.53–2.36)	0.522
FVC, % predicted	67.59 ± 18.19	59.40 ± 16.59	**0.009**
FEV_1_/FVC	62.5 (58.03–66.49)	57.3 (47.79–64.90)	**0.002**
pH	7.40 (7.35–7.45)	7.40 (7.36–7.42)	0.167
PaO_2_, mmHg	70 (54–86)	64 (54.5–75)	0.392
PaCO_2_, mmHg	42 (33–51)	47 (40–54)	0.143
Oxygen saturation, %	93 (90–96)	92 (88–95)	0.228
HCO_3_, mmol/L	26 (24–28)	27 (25–28)	0.410
Urea, mg/dL	31 (24–44)	37 (28–51)	0.184
Creatinine, mg/dL	0.8 (0.6–0.9)	0.8 (0.6–1.0)	0.897
AST, U/L	23 (16–30)	19 (14–25)	0.067
ALT, U/L	15.5 (12–26)	16 (12–23)	0.747
CRP, mg/L	24 (8–46)	45 (10–125)	**0.021**
WBC, ×10^3^/µL	9 (7–10)	10 (8–13)	**0.041**
Hemoglobin, g/dL	12.45 ± 2.0	13.04 ± 1.9	0.087
Platelets, ×10^3^/µL	258.5 (217–337)	279 (218–326)	0.940
Troponin T, pg/mL	16 (8–40)	13.5 (8–21)	0.367
pro-BNP, pg/mL	211 (65–1512)	460 (150–1453)	0.332
D-dimer, ng/mL FEU	1486 (1179–2590)	696 (456–1123)	**<0.001**

If the numeric variables fitted the normal distribution, they are presented as the mean ± SD; if not, they are presented as the median (IQR 25, 75) in this table. ALT, alanine aminotransferase; AST, aspartate aminotransferase; CRP, C-reactive protein; FEV_1_, forced expiratory volume in 1 s; FVC, forced vital capacity; HCO_3_^−^, bicarbonate; IQR, interquartile range; PaCO_2_, partial pressure of carbon dioxide; PaO_2_, partial pressure of oxygen; pro-BNP, pro-brain natriuretic peptide; PTE, pulmonary thromboembolism; SD, standard deviation; WBC, white blood cell count. Bold values indicate statistical significance (*p* < 0.05).

**Table 3:** Diagnostic performance of different D-dimer thresholds for imaging-confirmed pulmonary thromboembolism among patients who underwent confirmatory imaging (*n* = 87).

D-Dimer Threshold	Sensitivity, % (95% CI)	Specificity, % (95% CI)	PPV, % (95% CI)	NPV, % (95% CI)
**Fixed threshold of 500 ng/mL FEU**	94.7 (82.7–98.5)	16.3 (8.5–29.0)	46.8 (36.0–57.8)	80.0 (49.0–94.3)
**Age-adjusted threshold ^a^**	92.1 (79.2–97.3)	36.7 (24.7–50.7)	53.0 (41.2–64.6)	85.7 (65.4–95.0)
**Exploratory threshold of 1053 ng/mL FEU**	84.2 (69.6–92.6)	55.1 (41.3–68.1)	59.3 (46.0–71.3)	81.8 (65.6–91.4)

Data are presented as percentages with 95% confidence intervals. Among the 87 patients who underwent confirmatory imaging, 38 had imaging-confirmed PTE and 49 had imaging-confirmed absence of PTE. ^a^ The age-adjusted threshold was defined as 500 ng/mL FEU for patients aged ≤50 years and age × 10 ng/mL FEU for patients aged >50 years. CI, confidence interval; NPV, negative predictive value; PPV, positive predictive value; PTE, pulmonary thromboembolism.

**Table 4:** Univariable and multivariable logistic regression analyses of factors associated with diagnosed PTE.

Variable	Crude OR	95% CI	*p* Value	Adjusted OR	95% CI	*p* Value
D-dimer > 1053 ng/mL FEU	14.737	5.713–38.014	**<0.001**	20.317	6.804–60.664	**<0.001**
FEV_1_, % predicted	1.044	1.020–1.068	**<0.001**	1.023	0.995–1.052	0.113
FVC, % predicted	1.029	1.007–1.051	**0.010**	—	—	—
FEV_1_/FVC, %	1.064	1.020–1.110	**0.004**	—	—	—
CRP, per 10 mg/L increase	0.904	0.840–0.973	**0.007**	0.901	0.828–0.980	**0.015**
WBC, ×10^3^/µL	0.944	0.864–1.030	0.196	—	—	—
Female sex	3.613	1.707–7.649	**0.001**	4.401	1.548–12.511	**0.005**
Age, years	1.036	0.992–1.082	0.107	0.991	0.940–1.044	0.722

Crude ORs were obtained from univariable logistic regression analyses and adjusted ORs from the final multivariable model, which included D-dimer > 1053 ng/mL FEU, FEV_1_% predicted, age, CRP per 10 mg/L increase, and sex. The final model included all 181 patients, with no missing data for the included variables. The reference categories were D-dimer ≤ 1053 ng/mL FEU and male sex. Dashes indicate variables not included in the multivariable model. Bold values indicate statistical significance (*p* < 0.05). CI, confidence interval; CRP, C-reactive protein; FEV_1_, forced expiratory volume in 1 s; FVC, forced vital capacity; OR, odds ratio; PTE, pulmonary thromboembolism; WBC, white blood cell count.

## Data Availability

The data presented in this study are available from the corresponding author upon reasonable request, subject to institutional and ethical restrictions. The data are not publicly available due to patient privacy and ethical restrictions on the public sharing of individual-level clinical data.

## Supplementary Materials

The following supporting information can be downloaded at https://www.mdpi.com/article/10.3390/jcm15166394/s1, Table S1: Exploratory comparison of available spirometric parameters according to D-dimer testing status.

## Abbreviations

The following abbreviations are used in this manuscript:

PTE	Pulmonary thromboembolism
AECOPD	Acute exacerbation of chronic obstructive pulmonary disease
ED	Emergency department
COPD	Chronic obstructive pulmonary disease
CTPA	Computed tomography pulmonary angiography
VTE	Venous thromboembolism
PFT	Pulmonary function test
FEV_1_	Forced expiratory volume in 1 s
FVC	Forced vital capacity
CRP	C-reactive protein
pro-BNP	Pro-brain natriuretic peptide
V/Q	Ventilation/perfusion
SD	Standard deviation
ROC	Receiver operating characteristic
OR	Odds ratio
CI	Confidence interval
LTOT	Long-term oxygen therapy
