# Supplementary material for: Pulmonary Thromboembolism in Hospitalized Patients with Acute Exacerbation of COPD: Frequency, Predictors, and D-Dimer Performance
Source: J Clin Med. 2026 Aug 19;15(16):6394. doi: 10.3390/jcm15166394 (PMC13513376; doi:10.3390/jcm15166394)
Supplement: Supplementary file 1 [file jcm-15-06394-s001.zip › jcm-4466430-supplementary.pdf]

Variables	D-dimer testing not performed (n = 149)	D-dimer testing performed (n = 181)	p value
FEV1, L	0.915 (0.70–1.17)	1.08 (0.81–1.40)	**0.001**
FEV1, %	35.5 (25.93–44.44)	41.24 (33.33–55.27)	**<0.001**
FVC, L	1.76 (1.37–2.21)	1.95 (1.54–2.38)	**0.027**
FVC, %	54.46 ± 15.30	61.12 ± 17.21	**<0.001**
FEV1/FVC, %	54.3 (45.6–59.7)	58.5 (49.1–65.5)	**<0.001**

If numeric variables fitted the normal distribution, they were presented as mean ± SD; otherwise, they were presented as median (IQR 25,75). FEV_1, forced expiratory volume in 1 second; FVC, forced vital capacity; IQR, interquartile range; SD, standard deviation.

Table S1: Exploratory comparison of available spirometric parameters according to D-dimer testing status
